# Lipschütz Ulcer: A Rare Etiology Among Infants

**DOI:** 10.7759/cureus.76117

**Published:** 2024-12-21

**Authors:** Mariana S Pedro, Marta Caldas, Jorge Penas, Daniel Soares

**Affiliations:** 1 Pediatrics, Centro Hospitalar do Oeste, Unidade Caldas da Rainha, Caldas da Rainha, PRT

**Keywords:** infants, kissing lesions, lipschutz ulcer, nonsexually, no scar

## Abstract

An 11-month-old female patient presented to the pediatric emergency room, reporting a high fever and excessive crying. She began taking amoxicillin and clavulanic acid for acute otitis media five days prior. There was no record of trauma, suspected sexual abuse, or other medications involved. Physical examination showed a 7 mm ulcerative lesion on the left major labia and a symmetrical 5 mm violaceous lesion on the right major labia. Tests for herpesvirus, syphilis, HIV, Epstein-Barr virus, cytomegalovirus, and *Mycoplasma pneumoniae* all returned negative results. She was discharged with symptomatic treatment. She had no fever after one day, and the ulcers resolved four weeks later. During the one-month follow-up, the complete resolution of the ulcers confirmed the diagnosis of Lipschütz ulcer, a diagnosis based on exclusion. Lipschutz ulcer is a rare, self-limited condition that does not transmit sexually. It presents with the sudden appearance of painful, necrotic ulcers on the vulva or lower vagina. This condition mainly affects sexually inactive adolescent girls or young women and is extremely uncommon in children.

## Introduction

Vulvar ulceration in infants is a rare and often perplexing condition, posing significant diagnostic and therapeutic challenges for clinicians. Unlike genital ulcers in adults and adolescents, which are commonly associated with infectious agents (such as herpes simplex virus, syphilis, or human papillomavirus), autoimmune disorders (like Behçet's disease or lupus), or trauma (including sexual abuse or mechanical injury), the etiology of vulvar ulcers in infants remains poorly understood. The incidence of Lipschütz ulcer is unknown, and the average age at diagnosis reported was 16.6 years [1].

This rare presentation raises particular concerns, given the delicate age group and the need to rule out potential underlying conditions, including infections, trauma, sexual abuse, or dermatological disorders. First and foremost, the potential for serious underlying conditions must be carefully considered. Infections, both viral and bacterial, need to be thoroughly ruled out. Trauma is another significant concern, particularly in cases where there is suspicion of sexual abuse. Pediatricians should be vigilant in assessing any possible history of trauma, even in the absence of obvious signs. Dermatological disorders also play a crucial role in the differential diagnosis because conditions like dermatitis, eczema, or even rare autoimmune diseases that manifest as skin lesions can sometimes present as vaginal ulcers in infants [2,3].

As vulvar ulceration in infants is extremely rare, it is essential for healthcare providers to consider a broad differential diagnosis, including infections, trauma, and dermatological disorders, while approaching each case with caution and thoroughness. The rarity and complexity of this condition underscore the importance of a careful, evidence-based approach to diagnosis and management in order to ensure the best possible outcomes for affected infants [3,4].

## Case presentation

An 11-month-old female with no prior medical history presented to the pediatric emergency room with a high fever lasting for three days and a refusal to feed. Physical examination revealed erythema and bulging of the right tympanic membrane, with no other abnormalities. Acute otitis media was diagnosed, and amoxicillin was initiated. Two days later, due to persistent fever and the appearance of a cutaneous rash and a vulvar lesion, the patient was re-evaluated. There was no personal history of genital trauma, sexual abuse, drug intake, recent travels, skin disease, similar previous episodes, or autoimmune disease. There was also no family history of autoimmune diseases. 

The physical examination revealed a maculopapular rash on the trunk and edema and erythema of the left labia majora. Blood tests showed a white blood cell count of 13,400 WBC/μL (37.1% neutrophils) and a C-reactive protein level of 2.7 mg/dL (Table 1).

**Table 1:** Laboratory findings WBC: white blood count

Parameter	Result	Normal Range
Haemoglobin (g/L)	13.1	11.3–14.1
Platelets × 10^9^/L	250	205–553
WBC × 10^9^/L	13.4	6.0–17.0
Neutrophilis (× 10^9^/L)	4.97	1.5–8.5
C-reactive protein (mg/dL)	2.7	<0.5

Due to the appearance of the genital lesion, clavulanic acid was added to the antibiotic regimen, and a follow-up appointment was scheduled within two days.

**Figure 1:**
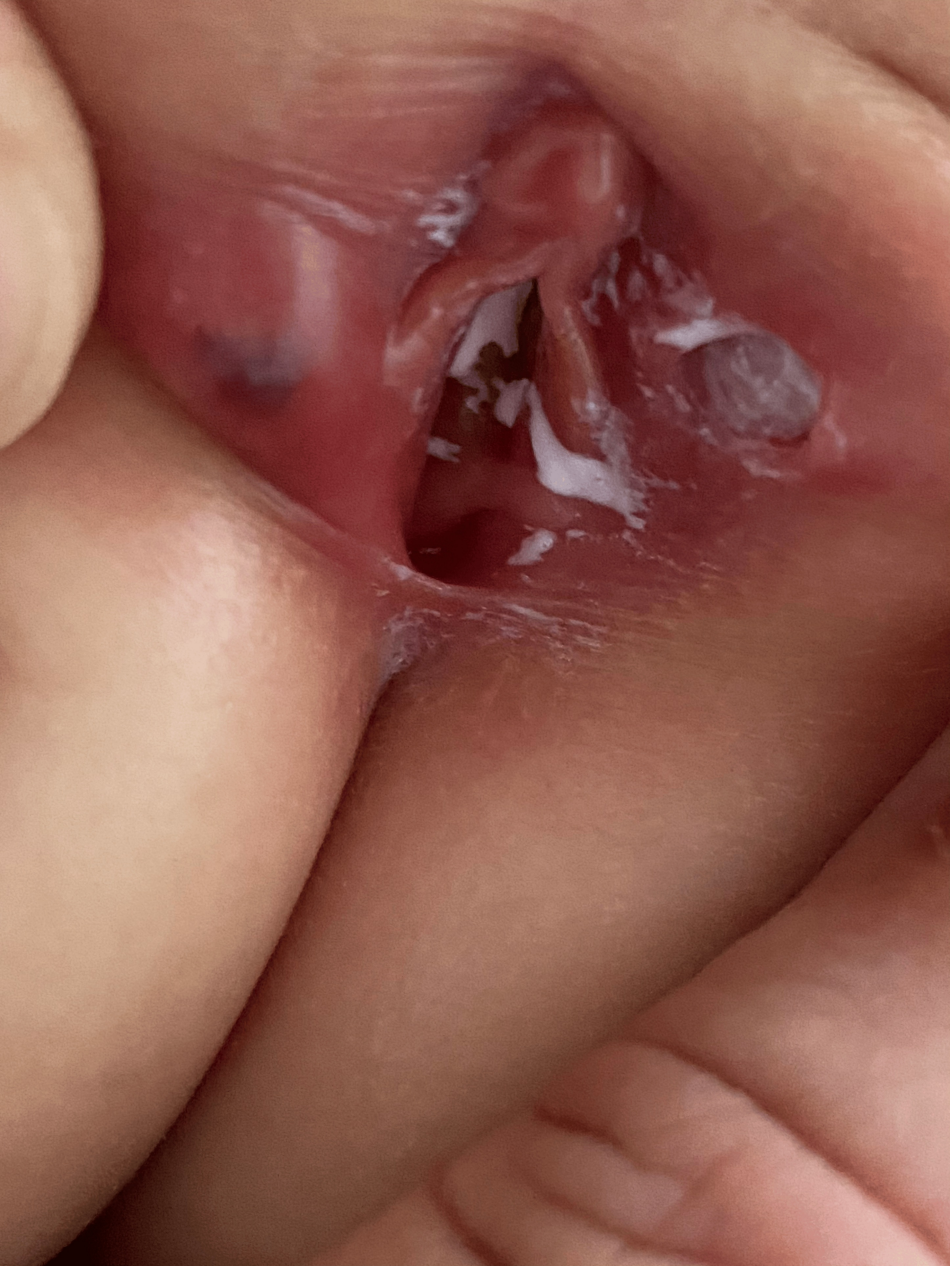
"Kissing ulcers" on major labia

During this period, the fever worsened, and the genital lesion also progressed. Upon re-evaluation, physical examination revealed a 7-mm painful ulcerative lesion on the left labia majora and a symmetrical 5-mm violaceous lesion on the right labia majora, known as "kissing ulcers" (Figure 1).

The diagnosis of Lipschütz ulcer was made, and the patient was discharged with analgesics, with a follow-up appointment scheduled within 48 hours. Serologies for herpesvirus, syphilis, HIV, Epstein-Barr virus, cytomegalovirus, and *Mycoplasma pneumoniae* all returned negative results.

**Figure 2:**
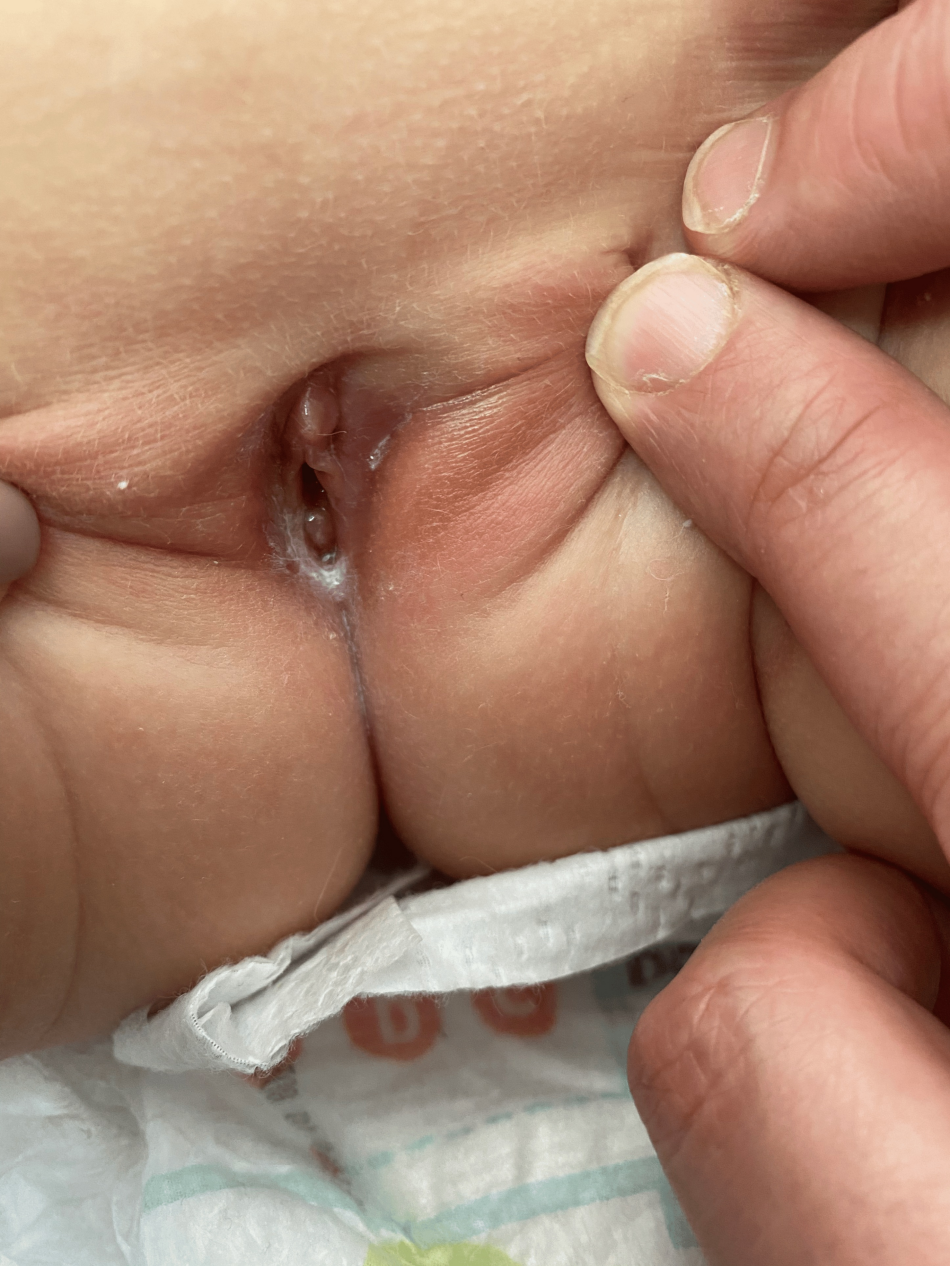
Complete resolution of lesion without scars

At the follow-up appointment, the mother reported that the patient had been afebrile since the previous visit, had an improved appetite, and that the genital lesion remained unchanged. Follow-up continued, and after three weeks, there was significant improvement in the genital lesion, with a 2-mm ulcerative lesion on the left labium majus and no erythema or edema of the right labium majus. One week later, the lesion had completely resolved without scarring (Figure 2).

## Discussion

Lipschütz ulcer corresponds to a rare, self-limited, and nonsexually transmitted condition that is often underdiagnosed and is characterized by the sudden or acute onset of painful, ulcerative lesions in the vulva, lower vagina, and/or perineum, with no prior history of sexual contact in girls and teenagers [2]. Lipschütz ulcer is extremely rare in children, with very few reports in the literature describing its occurrence in the first months of life, as seen in our patient [3,4].

The etiology remains uncertain, though it appears to be linked to infectious or idiopathic causes. However, in the majority of cases, no definitive association with an infection had been established.

Additional clinical symptoms such as fever, myalgia, odynophagia, and localized adenopathy typically accompany the onset of this genital lesion. These symptoms generally precede the appearance of vulvar ulcers by several days [1].

Lesions typically start as erythematous-violaceous vesicles, which evolve into painful, symmetric, and well-delimited ulcers with the fibrinous and necrotic centers. Lesions were usually one to about three and had a spontaneous resolution in two or three weeks, healing without scarring [1]. The right side is more commonly affected, but several reports describe bilateral symmetrical lesions, often referred to as "kissing ulcers" [4]. The appearance of this genital lesion is usually accompanied by other clinical features such as fever, general malaise, myalgia, odynophagia, and local adenopathy, which generally precede vulvar ulcers by several days [3].

The diagnosis is primarily clinical and is often made through exclusion. Biopsies are not required because the findings are nonspecific. However, due to the clinical nature of the diagnosis, it is essential to perform a careful differential diagnosis [2].

In addition to local hygiene, wound care, and pain management, providing an explanation and reassurance about the non-sexual transmission and self-limited nature of the condition is crucial [2].

## Conclusions

This case report describes an infant who presented with unexplained painful vulvar ulceration, highlighting the clinical course, diagnostic workup, and therapeutic approach of a Lipschütz ulcer. This is a diagnosis of exclusion, so it is important to rule out other causes of genital ulcers before making the diagnosis. To make the diagnostic process for this condition more efficient, it is important to keep a few key aspects in mind: it occurs only in children and adolescents without active sexual lives and is very rare in infants; a symmetrical appearance of painful ulcers ("kissing ulcers") is characteristic; healing without scarring is spontaneous under proper analgesia and wound care, and treatment is primarily supportive and focuses on reassurance, local hygiene, wound care, and pain management.
